# Parental attendance in two early-childhood training programmes to improve nurturing care: A randomized controlled trial

**DOI:** 10.1016/j.childyouth.2020.105418

**Published:** 2020-11

**Authors:** Rafaela Costa Martins, Adriana Kramer Fiala Machado, Yulia Shenderovich, Tâmara Biolo Soares, Suélen Henriques da Cruz, Elisa Raquel Pisani Altafim, Maria Beatriz Martins Linhares, Fernando Barros, Iná S. Santos, Joseph Murray

**Affiliations:** a Post-graduate Programme in Epidemiology, Federal University of Pelotas, Brazil; b Human Development and Violence Research Centre (DOVE), Federal University of Pelotas, Brazil; c Centre for Evidence-Based Social Intervention, Department of Social Policy and Intervention, Barnett House, 32 Wellington Square, Oxford OX1 2ER, United Kingdom; d Instituto Cidade Segura, Brazil; e Department of Neurosciences and Behavior, Ribeirão Preto Medical School, University of São Paulo, Brazil

**Keywords:** Parenting, Randomized controlled trial, Child, Brazil, Early intervention, Educational

## Abstract

Parent training programmes have significant potential to improve the quality of children's early environments and thereby their development and life-course outcomes. The aim of this study was to identify and explain the extent to which parents engaged in two group-based training programmes, offered to high-risk families enrolled in a randomized controlled trial study called PIÁ in Southern Brazil. The programmes were: (1) ACT: Raising Safe Kids, a 9-week programme aiming to reduce harsh parenting and maltreatment and improve positive parenting practices; (2) Dialogic book-sharing (DBS), an 8-week programme aiming to promote parental sensitivity and improve child cognitive development and social understanding. Of the 123 mothers randomly allocated to the ACT programme, 64.2% (n = 79) completed the course, and of 124 mothers allocated to DBS, 76.6% (n = 95) completed the course. After the interventions, mothers were very positive about the experience of both programmes but highlighted practical difficulties in attending. In adjusted regression analyses, only two variables significantly predicted ACT course completion (maternal age and distance between the intervention site and household); no significant predictor was found for DBS attendance. We conclude that although high completion rates are possible, there are important challenges to engaging parents of young children in training programmes, and practical difficulties occurring during training courses may be more important for attendance than baseline participant characteristics.

## Highlights

• ACT and DBS were well accepted by participants in a middle-income country (Brazil).
• Attendance rates of the interventions were high (ACT = 64.2%; DBS = 76.6%).
• Few variables predicted attendance rates in this study.

## 1 Introduction

Parent-training programmes aim to help parents build positive relationships with their children, use consistent, appropriate responses to child discipline problems, and stimulate optimal child development (Baker, Arnold, & Meagher, 2011). Such programmes have shown enormous potential to influence the quality of children's early environments and thereby their developmental and life-course outcomes. However, parental engagement in the training programmes is considered critical to their effectiveness (Nix et al., 2009, Weeland et al., 2017). Previous studies reported mixed results concerning participant attendance rates – about 39% to 81% (Annan et al., 2017, Garvey et al., 2006, Heinrichs et al., 2005, Shenderovich et al., 2018). Even among well-established parenting training programmes for which numerous trials show positive impacts on parenting and child outcomes, attendance rates have not always been as good as hoped – Triple P: 48.2%; The Incredible Years: 69.0%; Project SafeCare: 10.0% (Gershater-Molko et al., 2003, Leijten et al., 2017, Ozbek et al., 2018). If intervention up-take is poor under generally favourable conditions in research studies, this indicates a potentially serious problem for scale-up beyond the research setting, taking interventions to larger populations under less supervised and often less resourced conditions. As such, it is important to examine attendance rates and their determinants, as well as the impact of parental programmes for those who do actually adhere to them.

Many randomized trials have been conducted on the efficacy of parent-training programmes, for numerous parent and child outcomes. However, participant non-attendance in interventions can critically undermine study validity about programme effects, as well as carry implications for scale-up feasibility. If only a small proportion of allocated participants actually receive an intervention in a randomized trial, the true effects of the programme may be underestimated. This is because randomized trials should analyse data on an “intention to treat” basis to avoid bias (CONSORT, 2010) – comparing outcomes between the control group and *all* participants who were offered the intervention, regardless of whether they actually attended or completed the intervention. When trial participants fail to engage with interventions, researchers often resort to “per protocol” analyses (including only those that actually complete the intervention) with less reliable results since they were not randomized to that condition (CONSORT, 2010, Gupta, 2011). Furthermore, dropout decreases sample size and also statistical power (Baker et al., 2011). Therefore, advancing understanding of participation in parent training programmes is important to appropriately evaluate intervention effectiveness, as well for scale-up considerations.

Sociodemographic factors such as low socioeconomic status, ethnic minorities and single parenthood are some of the most consistent predictors of dropout in intervention programs (Bagner & Graziano, 2013, Baker et al., 2011, Chacko et al., 2016, Lavigne et al., 2010, Shenderovich et al., 2018). Parental psychological distress is also highlighted as of critical importance, affecting parents’ motivation to engage in an intervention, leading to lower attendance rate (Finan et al., 2018, Nock and Ferriter, 2005, Werba et al., 2006). Moreover, families that face problems like substance abuse or depression are less likely to engage in interventions (Bagner & Graziano, 2013). Among factors that have been found to support attendance in out of home interventions, offering transportation has been found to have a positive effect (Ingoldsby, 2010).

The current study of intervention attendance involves two group-based parenting programs known as ACT: Raising Safe Kids (ACT) and Dialogic Book-sharing (DBS). ACT is a short (9-week) low-cost programme with high cultural versatility developed by the American Psychological Association (APA). The aim of the programme is to help parents understand child development and raise their child without violence, using discussions and dramatizations. Attendance rates reported in previous studies of ACT range from 53% to 86% (Pontes, Siqueira, & Williams, 2019). ACT has been found to have some important benefits in terms of reducing harsh parenting and child conduct problems (Knox, Burkhart, & Hunter, 2010).

DBS is also a short (8-week) low-cost program, developed by the Mikhulu Trust in South Africa, and supported by the World Health Organization, in which parents learn how to interact sensitively and stimulate their child through book-sharing. The aim of the programme is to help parents support socio-cognitive and emotional development for their child. Attendance rates among prior studies using DBS methodology are around 88% (Vally, Murray, Tomlinson, & Cooper, 2015). DBS has been found to have some important benefits, in terms of sensitive and responsive parenting, and child attention, social understanding, and language development (Mikhulu Trust, 2019).

Although, participant attendance in parent-training programmes is seen as critical to their effectiveness in randomized controlled trials (Nix et al., 2009, Weeland et al., 2017), only a few such trials have been conducted in low-and middle-income countries, where it is estimated that around 43% of children are at risk for developmental delay (Lu, Black, & Richter, 2016). The current study was conducted in a middle-income country, Brazil (The World Bank, 2020), in the context of a randomized trial (PIÁ: the Pelotas Parenting Interventions for Aggression Trial). The main aim of the current analyses was to investigate the extent to which parents attended each of the two training programmes, which were offered to high-risk families (children with more aggressive behaviours in poorer families), and possible predictors of attendance rates. In addition, we describe post-intervention evaluations of the interventions by mothers enrolled in the trial. Programme effects will be reported separately.

## 2 Material and methods

The PIÁ study is a randomised controlled trial nested in a birth cohort study (the 2015 Pelotas Birth Cohort Study; Hallal et al., 2018). PIÁ is a three-arm trial, including two parenting interventions: 1) ACT: Raising Safe Kids, a group-based parenting programme aiming to reduce harsh parenting and maltreatment, and improve positive parenting practices; 2) Dialogic book-sharing (DBS), a group-based parenting programme aiming to promote parental sensitivity and improve child cognitive development and social understanding. A control group, not analysed in the current investigation of adherence rates, was also included in the study, including mothers receiving services as usual, without any extra, researcher-led interventions.

The 2015 Pelotas Birth Cohort Study and the PIÁ trial were approved by the Ethics Research Committee of the School of Physical Education from the Federal University of Pelotas and the Faculty of Medicine from the Federal University of Pelotas, respectively, under protocol numbers 26746414.5.0000.5313 and 2.602.769. All participants received full explanations about the study and signed an Informed Consent Form.

Data for the trial were collected between June 2018 and July 2019. The detailed methods of the trial are described elsewhere (Murray et al., 2019). The eligibility criteria for the trial were: a) participation in the 2015 Pelotas Birth Cohort Study follow-up when children were aged 2 years (95% of children recruited to the cohort were assessed at age 2); b) resident in the Pelotas city urban district; c) in the poorest 30% of the cohort families; d) children not considered as having signs of serious development delay (10% with lowest scores on development delay at age 2 years were excluded); e) children did not have low aggression scores (31% with lowest scores at age 2 years), because the trial aimed to prevent chronic child aggression; f) mothers and children did not have visual, speech or auditory impairment prohibiting participation in the interventions; g) the child was not a twin with a live sibling; h) the mother lives with the child at least 4 days a week; i) the mother reported potential availability to participate in 9 weekly parent training sessions, if invited – i.e. she did not indicate that critical work commitments or other issues would prohibit participating.

Recruitment to the study was conducted by a team phoning and house-calling mothers who had been identified as eligible from previous cohort assessments and inviting them to schedule an appointment for baseline assessment. For the ACT programme, mothers were invited to attend weekly 2-hour sessions, for 9 weeks. For DBS, mothers and their children were invited to attend one 1 ½ hour training sessions each week for 8 weeks. The week after baseline assessment, interventions began. On average, four weeks after the end of intervention, participants were invited for assessments again at the research centre.

After identifying eligible participants from the cohort and inviting sufficient numbers to participate (given power analysis for programme effectiveness; Murray et al., 2019), the trial sample included 369 mother–child dyads. Participant children were 2–3 years old at baseline. Immediately after baseline assessment, mother–child pairs were randomly assigned to one of the groups: 1) ACT: Raising Safe Kids, (n = 123), or 2) Dialogic book-sharing (DBS), (n = 121); or a Control group (n = 122), not considered further in the current analyses.

To encourage adherence to the two parenting programmes, a number of efforts were made by the trial research team and local government partner which implemented the interventions. First, for mothers allocated to an intervention group, the potential benefits of the intervention were emphasised face-to-face by a senior member of the research team after randomization; a video was shown about the benefits of the intervention related by a local mother who had previously completed the programme, and a leaflet was provided summarising this information. Second, to facilitate access, the programme group sessions were organised by neighbourhood and transport links, with mothers living in the same neighbourhood participating in the same group. Third, the timing of the sessions was organised by asking each mother when, during the week, she would be available to participate, and booking the group sessions according to when all could potentially participate. Fourth, telephone calls and messages were sent as reminders prior to each session. Fifth, childcare assistance was provided during the programme sessions, as well as snacks and financial assistance for transportation. In addition, for the first session, and on subsequent very rainy days, a van was organised to take mothers from their homes to the group sessions. At the end of the intervention, each mother received a certificate of participation and those completing the intervention were entered into a raffle (with two Android tablets as prizes). In addition, when mothers missed a particular programme session, they were invited to recuperate that session by joining in with another class, wherever possible.

Facilitator training in ACT was conducted via a 2-day workshop given by a postdoctoral psychologist and an ACT master trainer certified by the Violence Prevention Office of the APA (American Psychological Association). Facilitator training in DBS was delivered by David Jeffery of the Mikhulu Trust (www.mikhulutrust.org) in a 5-day course, with support from two supervisor researchers, who had also received prior DBS training. The training for each programme aimed to teach facilitators the content of the course, and how to deliver the course as well as possible, with high fidelity to the programme. Facilitators of ACT were senior school coordinators and social workers from municipal schools, and the facilitators of DBS were younger people working with vulnerable families in the Brazilian programme called PIM (*Primeira Infância Melhor*), which is a state home-visiting programme to support early child development among at-risk families.

ACT and DBS were implemented by Pelotas municipal government staff, under supervision by the research team, between July and December 2018. During the ACT sessions, a co-facilitator completed the session checklist ensuring that all the activities of the ACT programme had been conducted properly and, when necessary, reminding the facilitator of some activity or complementing the facilitator’s statements according to the programme guide. Additionally, weekly supervision sessions were held with between the ACT facilitators and a senior psychologist member of the research team, who also participated in the ACT training workshop. In ACT there was two facilitators for each session, and in DBS a single facilitator conducted the sessions. In both ACT and DBS, facilitators received weekly supervision from one of the research staff who had supported facilitator-training.

Participant attendance at each training session was recorded by the programme facilitator and communicated to the research team. We classified mothers as having completed the programmes if they participated in at least 7 out of 9 ACT sessions and 6 out of 8 DBS sessions, as defined by (ACT) and with (DBS) the programme developers. We examined possible correlates of programme attendance based on data collected prior to the trial (during the 24-month follow-up of the 2015 Pelotas Birth Cohort), as well as at the trial baseline assessment, and maternal evaluations of the interventions at the post-intervention assessment.

### 2.1 Potential correlates of programme attendance

From the 24-month cohort follow-up assessment, we examined the following variables as potential correlates of attendance in the parenting programmes: monthly family income (in quintiles), maternal education (categorized as 0–4, 5–8, 9 or more years of study), maternal relationship (without or with partner), household overcrowding (defined as the three or more people living in the house per room used to sleep), date of first intervention session (given seasonal [weather] changes through the study period), distance between training centre location and the mother’s home, calculated using Google Maps (for mothers who completed sessions at more than one centre, a weighted mean was calculated considering the number of sessions attended at each centre), and involvement in other interventions since pregnancy. As well as interventions in the PIÁ trial, we coded whether mothers had participated in three other local interventions, to test whether participation in multiple parenting programmes/trials might affect attendance in the programmes of the PIÁ trial. For this purpose, we measured participation in one intervention called PIM (“*Primeira Infância Melhor*” – a state government home-visiting programme to support child development among vulnerable families), and participation in one of two previous trials nested in the same birth cohort study: PAMELA (Domingues, Bassani, da Silva, & de Coll, 2015), a physical activity trial during gestation, and the Sleep Trial (Santos et al., 2016), implemented when children were 3 months old, providing guidance aimed to improve child sleep.

From the PIÁ trial baseline assessment, the following variables were considered possible correlates of programme attendance: maternal age (dichotomized as ≤ 25, >25 years); only child (yes or no); time spent with the study child during weekdays (categorized as 24 h or less), maternal intimate partner violence (scored positively on any of 13 questions from the WHO Violence against Woman questionnaire; García-Moreno, Jansen, Ellsberg, Heise, & Watts, 2005). We used the “Parenting and Family Adjustment Scales” (PAFAS), previously applied and tested in Brazil (Altafim et al., 2018, Santana, 2018), which evaluates parental practices and family adjustment in 40 items, to analyze harsh parenting (highest tercile on the coercive parenting subscale), positive and involved parenting (highest tercile on the parent–child relationship scale), and inconsistent discipline (highest tercile on the parental consistency sub-scale) (Sanders, Morawska, Haslam, Filus, & Fletcher, 2014) as possible predictors of intervention attendence. Child maltreatment was measured using the Juvenile Victimization Questionnaire (JVQ-R2), a 34 items questionnaire; maltreatment was coded positively if a positive answer was given to any of the 4 questions on lifetime maltreatment plus sexual assault by a known adult (Finkelhor, Hamby, Ormrod, & Turner, 2005). Child conduct problem was measured using the Strengths and Difficulties Questionnaire (SDQ), with a score > 3 considered the cut-off for elevated conduct problems (Fleitlich et al., 2000, Goodman et al., 2000); previously applied in Brazil (Salatino-Oliveira et al., 2016). For maternal alcohol consumption, we used the AUDIT (Alcohol Use Disorders Identification Test), a 10-item questionnaire for screening of alcohol disorders: a score > 7 indicates alcohol-related disorder (Saunders, Aasland, Babor, De La Fuente, & Grant, 1993); the questionnaire was previously translated for Portuguese (Mendéz, 1999). This questionnaire was added to the assessment battery shortly after the start of the study, so has more missing data than other questionnaires. We used the Edinburgh Postnatal Depression Scale (EPDS) for maternal depression screening. The questionnaire was validated for Brazil, comprises 10 items and the maximum score was 30; a score ≥ 8 was considered as the cut-off for maternal depression (Matijasevich et al., 2014). Last, for maternal perceived stress we used the Perceived Stress Scale (PSS), a 10-item questionnaire that was categorized as low, moderate and high stress levels (Cohen, Kamarck, & Mermelstein, 1983), and previously translated and validated in Brazil (Faro, 2015, Luft et al., 2007).

During the post-intervention phase, all mothers in the trial (100% retention) returned to the research centre for assessments, and those who had been allocated to ACT/DBS groups were asked about their perceptions of the programmes. Due to logistic problems, this questionnaire was not completed by all mothers. From these reports, we considered, as possible correlates of programme attendance, overall satisfaction with the intervention (from the question “How satisfied you are with the help you received?”), and perceived distance from the intervention centre (from the question “Was the intervention near my home?”). We also describe maternal perceptions of the programmes from the post-intervention assessments, in terms of quality, level of participation in discussions, difficulty level of sessions (measured as the average of difficulty indicated for each session in which the mother participated), whether the mother would recommend the intervention to others, and if she plans to use what she has learned. Hence, although we do not have an observational measure for each mother of positive engagement in the sessions she went to, these reports provide summary information of maternal experiences and engagement of participating in the programmes.

### 2.2 Data analytic plan

Descriptive analyses were conducted to show relative and absolute frequencies of attendance in ACT and DBS according to possible predictors. Associations were assessed by Fisher’s exact test to obtain p-values, using a statistical significance level of 5%. Crude and adjusted analyses of associations between programme completion (yes or no) and the predictors were analysed using logistic regression to obtain the odds ratio; for supplementary analyses of the number of sessions attended, we used Poisson regression with robust variance to obtain incidence rate ratio. Statistical analyses were performed using Stata software version 15.0 (StataCorp LP, College Station, USA). Satisfaction data are reported separately for ACT and DBS.

We used a seven-level hierarchical conceptual model to structure the adjusted analysis, based on an ecological model, considering first distal family sociodemographic factors, and adding in subsequent models more proximal factors closer to the child, as well as finally including practical variables related to the current and past interventions and trials. In the first level, we included the following variables: maternal age, maternal schooling, maternal relationship, family income, only child, overcrowded houses and time spent with the child during weekdays. In the second level, we inserted the behavioural maternal variables (stress and depression). In the third level, domestic violence and child maltreatment were added to the model. In the fourth level, parenting variables were added (harsh parenting, positive and involved parenting and inconsistent discipline). In the fifth level, variables indicating participation in PIM and participation in any other trial were included. In the sixth level, child conduct problems were added; and in the last level, we added variables indicating the distance between home and the programme centre, satisfaction with the programme and date of first session. Both perceived and calculated distance were inserted to the model because they were not collinear (VIF < 10). In the adjusted analyses, the variables were inserted into the model using backwards selection, each level at a time, excluding those variables with p < 0.20.

## 3 Results

**Fig. 1:**
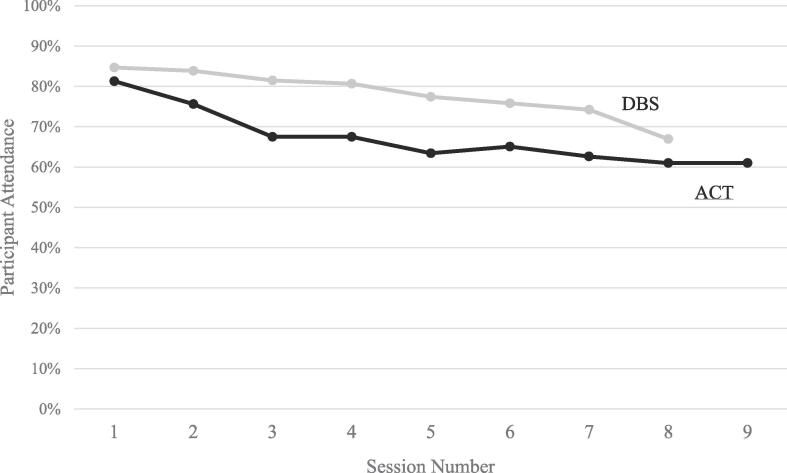
Percentage of participants attending each session in DBS and ACT.

Of the 123 mothers invited to participate in the ACT programme, 64.2% (n = 79) completed the intervention, and of the 124 mothers invited to participate in DBS, 76.6% (n = 95) completed the intervention. This overall difference in completion rates between the two interventions was significant (DBS > ACT, p < 0.05). Also, compared to DBS, more mothers invited to participate in ACT did not even start the course – 17.1% for ACT versus 14.5% for DBS, but this difference was not significant (p = 0.61). Among the 39 mothers who did not start either course, the most commonly reported reason given was job related (n = 15), followed by lack of interest (n = 7). As shown in Fig. 1, mothers allocated to ACT also had a higher rate of decline in participating in the subsequent two sessions compared to the DBS group. From the third session onwards, participation in the ACT group stabilized at about 63%. In the DBS group, there was a sustained smooth decline in maternal attendance at the course sessions, from session number 1 to session number 7.

Of all mothers who were invited to participate in ACT or DBS (n = 247), the majority were > 25 years old (61.9%), with 5–8 years of schooling (48.2%), and with a partner (71.1%). Most mothers (60.8%) spent most of their time looking after their children. Most did not have any alcohol disorder (96.6%), but just over half screened positive for depression (53.9%), and most reported moderate levels of stress (68.7%). Regarding parenting practices, most mothers reported relatively positive and involved parenting practices (78.9%), with few reporting inconsistent discipline (19.9%), or harsh parenting practices (26.4%); however, 16.7% of children were reported to have suffered some form of maltreatment. Most children (71.7%) scored high on the SDQ conduct problem scale.

As shown in Table 1, few maternal or child characteristics were significantly associated with programme completion in ACT. Completion of the course was more common among younger mothers, those living nearer to the intervention site and those satisfied with the programme in post-intervention assessment interviews. Regarding DBS, no variable was significantly associated with programme completion.

**Table 1:** Correlates of completing DBS and ACT parenting programmes in Pelotas, Brazil.

	ACT			DBS		
	N	Completed Intervention (%)	p-value	N	Completed Intervention (%)	p-value
Maternal age			0.01			0.82
≤25	58	44 (75.9)		36	27 (75.0)	
>25	65	35 (53.9)		88	68 (77.3)	

Maternal education (years)			0.76			0.46
0–4	18	13 (72.2)		22	15 (68.2)	
5–8	58	37 (63.8)		61	49 (80.3)	
9+	47	29 (61.7)		41	31 (75.6)	

Maternal relationship			0.54			0.48
Without partner	34	22 (64.7)		37	29 (78.4)	
With partner	88	56 (63.6)		87	66 (75.9)	

Family income (tertile)			0.65			0.76
1° (poorest)	43	30 (69.8)		40	31 (77.5)	
2°	35	22 (62.9)		48	38 (79.2)	
3° (richest)	45	27 (60.0)		36	26 (77.2)	

Only child			0.13			0.51
Yes	54	39 (72.2)		46	37 (80.4)	
No	69	40 (58.0)		78	58 (74.4)	

Overcrowding			0.26			0.39
<3 individuals per room	53	31 (58.5)		76	56 (73.7)	
≥3 individuals per room	70	48 (68.6)		48	39 (81.3)	

Time mother spends with child during weekdays			0.18			0.20
<24 h	49	35 (71.4)		47	33 (70.2)	
All the time	72	42 (58.3)		77	62 (80.5)	

Maternal alcohol use			1.00			0.64
No	98	62 (63.3)		101	76 (75.3)	
Yes	1	1 (100.0)		6	4 (66.7)	

Maternal depression			1.00			0.83
No	61	39 (63.9)		53	40 (75.5)	
Yes	62	40 (64.5)		71	55 (77.5)	

Maternal perceived stress			0.69			0.77
Low	25	15 (60.0)		41	30 (73.2)	
Moderate	93	61 (65.6)		76	59 (77.6)	
High	4	2 (50.0)		7	6 (85.7)	

Positive and involved parenting			0.10			1.00
No	24	19 (79.2)		28	22 (78.6)	
Yes	98	59 (60.2)		96	73 (76.0)	

Harsh parenting			0.09			0.23
No	91	54 (59.3)		90	66 (73.3)	
Yes	31	24 (77.4)		34	29 (85.3)	

Inconsistent discipline			0.49			0.60
No	98	61 (62.2)		99	77 (77.8)	
Yes	24	17 (70.8)		25	18 (72.0)	

Child maltreatment			0.47			1.00
No	101	66 (65.4)		104	80 (76.9)	
Yes	21	12 (57.1)		20	15 (75.0)	

Child conduct problems			0.51			0.18
Normal	29	17 (58.6)		41	28 (68.3)	
High	94	62 (66.0)		83	67 (80.7)	

Maternal intimate partner violence			0.98			0.60
Without partner	34	22 (64.7)		37	29 (78.4)	
No	65	41 (63.1)		58	42 (72.4)	
Yes	23	15 (65.2)		29	24 (82.8)	

Participated in PIM			0.65			0.15
No	97	61 (62.9)		91	73 (80.2)	
Yes	26	18 (69.2)		33	22 (66.7)	

Participated in any other trial			1.00			0.79
No	93	60 (64.5)		101	78 (77.2)	
Yes	30	19 (63.3)		23	17 (73.9)	

Month of the first session			0.18			0.07
July	31	27 (87.1)		28	22 (78.6)	
August	34	23 (67.7)		49	34 (69.4)	
September to November	37	29 (78.4)		44	39 (88.6)	

Perceived distance from DBS/ACT centre			0.06			0.53
Far	55	39 (70.9)		51	47 (87.0)	
Near	46	40 (87.0)		54	47 (92.2)	

Distance from DBS/ACT centre (km)			0.15			0.50
≤1.5	47	36 (76.6)		35	29 (82.9)	
1.6–3.0	33	19 (59.6)		40	28 (70.0)	
3.1–5.0	25	14 (56.0)		29	24 (82.8)	
>5	18	10 (55.6)		19	14 (73.7)	

Maternal satisfaction with DBS/ACT			0.02			0.12
Unsatisfied	0	0 (0.0)		1	1 (100.0)	
Satisfied	29	18 (62.1)		36	29 (80.6)	
Very satisfied	72	61 (84.7)		68	64 (94.1)	
**Total**	**123**	**79 (64.2)**		**124**	**95 (76.6)**	

Missing data vary by variables, and the number of participants with missing data were as follows for ACT: 2 for time mother spends with child during weekdays; 24 for maternal alcohol use; 1 for maternal perceived stress; 1 for positive and involved parent; 1 for inconsistent discipline; 1 for child maltreatment; 1 for maternal intimate partner; 22 for distance from ACT centre; 22 for maternal satisfaction with ACT. For the DBS group the number of participants with missing data were as follows: 17 for maternal alcohol use; 19 for perceived distance from DBS centre; 19 for maternal satisfaction with DBS.

Table 2 shows crude and adjusted logistic regression results for the potential predictors of completing each intervention. Alcohol consumption was subtracted of the raw and adjusted analysis, because only few mothers reported that behaviour. Also, for the maternal satisfaction with each program, we used only the categories “satisfied” and “very satisfied” due to the low number of respondents to “unsatisfied”. After adjustment, only three variables remained significant in the model for ACT (maternal age, distance between the intervention site and household, and satisfaction with the intervention). In DBS, no predictor was significant in the adjusted model. In the supplementary table, we also analysed possible predictors of the number of sessions that the mother attended for each programme (as count variables in Poisson regression), and similar results were obtained.

**Table 2:** Crude and adjusted analysis of rates of ACT and DBS course completion, according to maternal and other characteristics, in the Pelotas PIÁ Trial.

	ACT		DBS	
	Crude OR (95%CI)	Adjusted OR (95%CI)	Crude OR (95%CI)	Adjusted OR (95%CI)
	**First level**			
Maternal age				
≤25	1.00	1.00	1.00	1.00
>25	**0.37 (0.17**–**0.81)**	**0.38 (0.17**–**0.83)**	1.13 (0.46–2.80)	1.55 (0.53–4.56)

Maternal education (in years)				
0–4	1.00	1.00	1.00	1.00
5–8	0.68 (0.21–2.17)	0.43 (0.12–1.49)	1.91 (0.64–5.71)	2.30 (0.73–7.17)
9+	0.62 (0.19–2.03)	0.45 (0.13–1.57)	1.45 (0.46–4.55)	1.50 (0.44–5.10)

Maternal relationship				
Without partner	1.00	1.00	1.00	1.00
With partner	0.96 (0.42–2.19)	1.44 (0.58–3.57)	0.87 (0.34–2.18)	0.71 (0.26–1.92)

Income (tertile)				
1° (poorest)	1.00	1.00	1.00	1.00
2°	0.73 (0.29–1.89)	0.61 (0.21–1.74)	1.10 (0.40–3.05)	1.07 (0.37–3.08)
3° (richest)	0.65 (0.27–1.57)	0.60 (0.22–1.63)	0.76 (0.27–2.14)	0.79 (0.27–2.33)

Only child				
Yes	1.00	1.00	1.00	1.00
No	0.53 (0.25–1.14)	0.77 (0.31–1.90)	0.71 (0.29–1.72)	0.65 90.26–1.60)

Overcrowded houses				
<3 individuals per room	1.00	1.00	1.00	1.00
≥3 individuals per room	1.55 (0.74–3.26)	1.30 (0.59–2.89)	1.55 (0.64–3.76)	1.40 (0.56–3.51)

Time spent with the children during weekdays				
<24 h	1.00	1.00	1.00	1.00
All the time	0.56 (0.26–1.22)	0.53 (0.24–1.18)	1.75 (0.76–4.07)	1.75 (0.76–4.07)

	**Second level**			
Maternal depression				
No	1.00	1.00	1.00	1.00
Yes	1.03 (0.43–2.14)	0.96 (0.41–2.23)	1.12 (0.48–2.58)	0.96 (0.35–2.64)

Maternal perceived stress				
Low	1.00	1.00	1.00	1.00
Moderate	1.27 (0.51–3.15)	1.20 (0.47–3.08)	1.27 (0.53–3.06)	1.28 (0.53–3.10)
High	0.67 (0.08–5.54)	0.50 (0.05–4.60)	2.20 (0.24–20.40)	2.52 (0.27–23.83)

	**Third level**			
Maternal intimate partner violence				
Without partner	1.00	1.00	1.00	1.00
No	0.93 (0.39–2.21)	1.17 (0.45–3.03)	0.72 (0.27–1.91)	0.61 (0.22–1.68)
Yes	1.02 (0.34–3.10)	1.06 (0.32–3.47)	1.32 (0.38–4.58)	1.17 (0.33–4.13)

Child’s maltreatment				
No	1.00	1.00	1.00	1.00
Yes	0.71 (0.27–1.84)	0.53 (0.19–1.49)	0.90 (0.30–2.73)	0.72 (0.22–2.30)

	**Fourth level**			
Positive and involved parenting				
No	1.00	1.00	1.00	1.00
Yes	2.51 (0.87–7.29)	2.16 (0.72–6.46)	1.16 (0.42–3.20)	1.22 (0.43–3.47)

Harsh parenting				
No	1.00	1.00	1.00	1.00
Yes	2.35 (0.92–6.02)	1.64 (0.59–4.57)	2.11 (0.73–6.08)	2.00 (0.69–5.80)

Inconsistent discipline				
No	1.00	1.00	1.00	1.00
Yes	1.47 (0.56–3.89)	1.03 (0.36–2.92)	0.74 (0.27–1.98)	0.61 (0.22–1.71)

	**Fifth level**			
Participated in PIM				
No	1.00	1.00	1.00	1.00
Yes	1.33 (0.52–3.36)	1.02 (0.38–2.77)	0.49 (0.20–1.20)	0.51 (0.21–1.24)

Participated in any trial				
No	1.00	1.00	1.00	1.00
Yes	0.95 (0.40–2.24)	1.14 (0.47–2.78)	0.84 (0.30–2.37)	0.92 (0.32–2.66)

	**Sixth level**			
Child's conduct problems				
Normal	1.00	1.00	1.00	1.00
High	1.37 (0.58–3.21)	1.09 (0.44–2.73)	1.94 (0.83–4.57)	1.87 (0.79–4.42)

	**Seventh level**			
Month of the first session				
July	1.00	1.00	1.00	1.00
August	0.31 (0.09–1.11)	0.50 (0.10–2.42)	0.62 (0.21–1.84)	3.62 (0.59–22.25)
September to November	0.54 (0.15–1.99)	0.89 (0.18–4.37)	(0.58–7.78)	1.99 (0.35–11.16)

Perceived distance from DBS/ACT centre				
Far	1.00	1.00	1.00	1.00
Near	2.74 (0.97–7.71)	1.92 (0.51–7.29)	1.75 (0.48–6.38)	0.79 (0.13–4.64)
Distance from DBS/ACT centre (km)				
≤1.5	1.00	1.00	1.00	1.00
1.6–3.0	0.42 (0.16–1.09)	**0.15 (0.03**–**0.78)**	0.48 (0.16–1.46)	5.33 (0.58–49.03)
3.1–5.0	0.39 (0.14–1.10)	**0.10 (0.02**–**0.54)**	0.99 (0.27–3.66)	3.37 (0.41–27.50)
>5	0.38 (0.12–1.21)	**0.13 (0.02**–**0.83)**	0.58 (0.15–2.23)	0.78 (0.11–5.38)

Satisfaction with the intervention				
Satisfied	**1.00**	**1.00**	**1.00**	1.00
Very satisfied	3.39 (1.26–9.10)	5.32 (1.59–17.80)	3.86 (1.05–14.24)	4.98 (1.26–19.74)

95%IC: 95% confidence interval; OR: odds ratio; significant results are in bold; alcohol consumption and the category “less than satisfied” were excluded from this analysis due to the small sample sizes. In adjusted analyses, variables are adjusted for all other variables in the same and preceding levels.

On returning for post-intervention assessment, 114 of the mothers in the ACT group reported on their experience of ACT, with 43.0% (n = 49) saying they had found it difficult to attend the sessions. Of the 49 mothers, the following difficulties were cited: work issues (26.6%), lack of time (26.6%), no one available to care for the children (16.3%), health issues (12.2%), other family commitments (8.2%), distance to the programme centre (2.0%), pregnancy (2.0%), and a combination of multiple problems (6.1%). In DBS, out of 112 mothers reporting on their experience with that programme, 11.6% reported they had found it difficult to attend because of time issues (other possible difficulties were not asked about for DBS).

As shown in Table 3, in post-intervention assessment, most mothers, in both programme groups, reported that the quality of the instruction received had been “very good” (76.2% in DBS and 84.1% in ACT), and that they were satisfied with the programme experience (64.8% in DBS and 71.3% in ACT). Nearly all reported participating in the group discussions (95.2% in DBS and 91.0% in ACT), and the vast majority found the programme content easy to follow (91.3% in DBS and 84.2% in ACT). All mothers reported that they would recommend the programmes to a friend and all mothers in DBS and 99% of mothers in ACT said that they planned to continue to use the content learned in the programmes. When asked about the distance of the programme centre from their homes, 51.4% of DBS and 54.5% of ACT participants reported that it was far. Regarding financial assistance for food and transportation, around 71.3% of DBS and 77.1% of ACT participants said this was very important to help them participate in the sessions. There was no difference between ACT and DBS related to any variable analysed in Table 3.

**Table 3:** Maternal post-intervention perceptions about DBS and ACT programmes among mothers who participated in at least one session of each intervention.

	ACT (n = 102)		DBS (n = 106)	
	n (%)	CI95%	n (%)	CI95%
Overall quality*^,^†				
Very good	85 (84.1)	75.6–90.1	80 (76.2)	67.0–83.4
Good	15 (14.9)	9.1–23.3	24 (22.9)	15.7–32.0
Less than good	1 (1.0)	0.1–6.8	1 (0.9)	0.1–6.6

Overall satisfaction*^,^†				
Very satisfied	72 (71.3)	61.6–79.3	68 (64.8)	55.1–73.4
Satisfied	29 (22.7)	20.7–38.4	36 (34.3)	25.8–44.0
Less than satisfied	0 (0.0)	–	1 (0.9)	0.1 – 6.6

Participated in discussions**^,^†				
Very much	91(91.0)	83.5–95.3	100 (95.2)	89.0–98.0
A bit	9 (9.0)	4.7–16.5	3 (2.9)	0.9–8.6
Not at all	0 (0.0)	–	2 (1.9)	0.5–7.4

Would recommend the programme to a friend*^,^††				
Definitely	71 (70.3)	60.6–78.5	63 (60.6)	50.8–69.6
Should make an effort	21 (20.8)	13.9–29.9	33 (31.7)	23.4–41.4
If they have time	9 (8.9)	4.7–16.4	8 (7.7)	3.9–14.7
Should not participate	0 (0.0)	–	0 (0.0)	–

Financial assistance was important*^,^†				
Not at all	1 (1.0)	0.1–6.8	2 (1.9)	0.5–7.4
A little	28 (27.7)	19.8–37.3	22 (21.0)	14.1–29.9
Very important	72 (71.3)	61.6–79.3	81 (77.1)	68.0–84.3

Difficulty level of the intervention*^,^††				
Easy	85 (84.2)	75.6–90.1	95 (91.3)	84.1–95.5
Difficult	16 (15.8)	9.9–24.4	9 (8.7)	4.5–15.9

Note: These results are based on 102 ACT mothers and 106 DBS mothers who completed a post-intervention questionnaire about the experience of the programme they participated in due to logistic problems in the beginning of data collection.

* One missing value for ACT;

** Two missing values for ACT;

† One missing value for DBS;

†† Two missing values for DBS

## 4 Discussion

Although parent-training programmes have great potential to improve the caregiving environment and thereby support child development, parent engagement with such programmes is critical to effectiveness. In the context of a randomized trial evaluating two group-based parent-training programmes for mothers of young children, we examined the extent to which mothers who were invited to participate, attended each programme, and their self-reports of their experiences with the programmes. For both programmes (ACT and DBS), a significant proportion (15–20%) of mothers who were invited to participate did not start the course, despite significant efforts to relate the potential benefits of the programme for children, and to make attendance as easy as possible, in terms of programme location, support such as transportation, and timing of sessions. A parent-training prevention programme in the United States addressing child conduct problems identified a similar pattern of non-participation starting in the first session (Baker et al., 2011).

After the start of the parenting programmes in the current study, participation in the subsequent sessions of the DBS programme was higher than in the ACT programme. The following hypotheses might explain this difference in adherence between the two programmes, despite their broadly similar format (short, group-based, free, weekly training sessions): a) the content of ACT programme (aiming to address harsh parenting) may have been less attractive to mothers than the DBS programme which focuses on a positive activity of book-sharing; b) the additional time required for ACT sessions might have been a deterrent (2 h rather than 1½ hours in DBS; c) the facilitators had different characteristics – ACT was implemented by school coordinators and social workers, while DBS was implemented by people working with vulnerable families in the health sector; d) ACT is implemented with mothers only (children are not involved), and it is recommended that children do not participate in ACT sessions (although childcare was provided during ACT sessions in the current trial); the fact that DBS requires child participation may have helped with maternal attendance in DBS. Additionally, regarding the ACT program, the bond between the facilitator and the group is fundamental for the content of the programme to be covered adequately. This relationship is strengthened throughout the sessions and may be related to the participation attendance stabilization from the third session on.

After the initial programme sessions, each programme had a somewhat different pattern of attendance – with 64% of mothers completing the ACT programme, and 77% of mothers completing the DBS course. The final retention rate in the ACT programme was similar to a previous RCT in Brazil (66%; Altafim & Linhares, 2019), and within the range of rates observed in other ACT studies internationally, ranging from 53% to 86% (Pontes et al., 2019). Regarding DBS, the rate of completing all eight sessions was higher in a study in South Africa (88% against 64% in our study) (Vally et al., 2015). These findings indicate particular difficulty in engaging all mothers at the beginning of a programme and, depending on the programme, the need for extra efforts to maximize adherence especially during the first few sessions.

Mothers reported a number of difficulties that hampered attending all programme sessions, many of which were similar to those reported in a previous study of the ACT programme in Brazil. The main dropout reasons mentioned by participants were difficulties with the time of the meetings (even though these had been organised to fit with mothers’ schedules at the start of our study), work issues, and health and family issues (Altafim, Pedro, & Linhares, 2016). Given the efforts that were made in the current study to engage mothers in both programmes, and make participation as practical and attractive as possible, it is a concern that these types of problems may have a greater impact on attendance when such programmes are implemented at scale. Another element that could affect attendance is social desirability, since people may not feel comfortable saying they don’t like the programme or the group, but it’s acceptable to say they are busy (Zemore, 2012).

The possible predictors of programme completion we examined in this study were selected based on relevance to the programme type and prior studies of programme adherence (Shenderovich et al., 2018). An interesting finding of our study was that very few maternal or child characteristics predicted ACT or DBS attendance. After adjustment, the only three predictors of ACT attendance were maternal age, household distance from intervention’s site and maternal satisfaction with the intervention; in DBS there was no significant predictor. Regarding both programmes, mothers expressed high degrees of satisfaction with the courses, and the most satisfied mothers had higher attendance rates (for ACT only).

The lack of more predictors of programme completion might be explained by the efforts made in this trial to increase programme attendance, reducing the impact of some otherwise important variables. Another possible reason for the many null results is the relatively homogenous group selected for the interventions (i.e. high-risk families defined by high poverty levels and with more difficult child behaviour); this might have reduced the variability in otherwise potentially important predictors. Perhaps most plausibly, the most important determinants of attendance might be more dynamic (e.g. unpleasant weather during a particularly cold winter, illness in the family, or other practical difficulties), rather than the more static information captured at fixed time-points (prior and after interventions) in our trial. It will be important for future studies to collect more detailed data on such dynamic processes occurring through programme implementation, and to identify whether similar factors impede, or can encourage, programme participation across different social and cultural contexts.

Results across this and other studies examining predictors of adherence to parenting programmes are quite mixed. Although a study conducted in the United States (Knox & Burkhart, 2014) found that younger parents had lower retention rates in the ACT programme than older parents, and a Brazilian study (Altafim & Linhares, 2019) did not find differences by age, we found that younger parents actually adhered better to the ACT programme. A previous study in Brazil (Pedro, Altafim, & Linhares, 2017) examined retention rates for ACT based on socioeconomic status, showing that the retention rate was lower in a low-income group (51%) than in a higher income group (79%). Baker and colleagues (2011) also found that high income families had higher adherence rates than low income families (83% vs. 38%; p < 0.01; Baker et al., 2011). Finally, a metanalysis conducted by Reyno was in line with these individual results about drop out and socioeconomic status. (Reyno & McGrath, 2006). Because the current study was conducted with mothers of low socioeconomic status (family income in poorest tercile in the cohort), we would not expect to find that family income would explain attendance differences so strongly in our study, and no differences were observed.

Both interventions were evaluated very positively by the participants, regarding the quality, facility, satisfaction, and possibility of recommending to a friend. These positive perceptions demonstrate that the participants of both parenting programs had a good acceptance of the interventions. As highlighted in the literature, there has been a rapid global transporting of evidence-based parenting programs to different countries from where they were originally developed, and little is known about factors that can influence their implementation in new contexts (Gardner, Montgomery, & Knerr, 2016). Therefore, considering that both programs were developed in other countries, the high acceptability of them in a Brazilian population, and relatively high adherence rates, show that they are promising interventions for possible public policies.

This study has some limitations. We examined what may be considered more distal family and child characteristics as potential correlates of programme adherence, and it may be that more proximal factors (e.g. facilitator characteristics or dynamic factors during the interventions) are more helpful in explaining variation in attendance. We were not able to assess whether the quality of implementation by each facilitator correlated with adherence rates because many mothers received programme sessions from multiple facilitators (when they missed sessions or needed to change schedule). Of course, the results may not generalize to other social settings, or other interventions. Another issue is that for two variables (mothers’ perceptions about the interventions and maternal alcohol use) there was a significant proportion of missing data, limiting conclusions about the importance of these two variables.

## 5 Conclusion

Our study contributes to the scarce literature of predictors of engagement in parenting programmes in randomized controlled trials in middle- income countries. This study can help the planning of future trials and public policies scaling up parent-training programmes, by taking into consideration the strategies we took to increase attendance to reasonably high levels, and the barriers to programme-engagement highlighted by mothers in the study. Finally, research is needed to better understand dynamic issues involved in programme participation during its implementation.

## Funding

This work was supported by the 10.13039/100010269Wellcome Trust Foundation [Investigator Award to JM – 210735_Z_18_Z], 10.13039/501100011318Fondation Botnar (Project 6260), Pelotas City Hall, 10.13039/501100002322*Coordenação de Aperfeiçoamento de Pessoal de Nível Superior - Brasil (CAPES)* – Finance Code 001, and the UKRI GCRF Accelerating Achievement for Africa’s Adolescents Hub: ES/S008101/1.

## CRediT authorship contribution statement

**Rafaela Costa Martins:** Conceptualization, Methodology, Formal analysis, Investigation, Data curation, Writing - original draft, Writing - review & editing. **Adriana Kramer Fiala Machado:** Methodology, Formal analysis, Data curation, Writing - original draft, Writing - review & editing. **Yulia Shenderovich:** Writing - original draft, Writing - review & editing. **Tâmara Biolo Soares:** Investigation, Writing - original draft, Writing - review & editing. **Suélen Henriques da Cruz:** Investigation, Writing - original draft, Writing - review & editing. **Elisa Raquel Pisani Altafim:** Writing - original draft, Writing - review & editing. **Maria Beatriz Martins Linhares:** Writing - review & editing. **Fernando Barros:** Writing - review & editing. **Iná S. Santos:** Writing - review & editing, Supervision. **Joseph Murray:** Conceptualization, Methodology, Resources, Writing - review & editing, Supervision, Funding acquisition.

## Declaration of Competing Interest

The authors declare that they have no known competing financial interests or personal relationships that could have appeared to influence the work reported in this paper.
