# Supplementary material for: Parental attendance in two early-childhood training programmes to improve nurturing care: A randomized controlled trial
Source: Child Youth Serv Rev. 2020 Nov;118:105418. doi: 10.1016/j.childyouth.2020.105418 (PMC7607382; doi:10.1016/j.childyouth.2020.105418)
Supplement: Supplementary data 1 [file mmc1.docx]

**Supplementary Table.** Crude and adjusted analysis of the number of ACT and DBS sessions, according to maternal and other characteristics, in the Pelotas PIÁ Trial

|  | **ACT** | | **DBS** | |
| --- | --- | --- | --- | --- |
|  | **Crude IRR (95%CI)** | **Adjusted IRR (95%CI)** | **Crude IRR (95%CI)** | **Adjusted IRR (95%CI)** |
|  | **First level** | | | |
| Maternal age |  |  |  |  |
| ≤ 25 | **1.00** | **1.00** | 1.00 | 1.00 |
| >25 | **0.72 (0.58 - 0.89)** | **0.72 (0.58 - 0.89)** | 1.01 (0.84 - 1.20) | 1.11 (0.91 – 1.35) |
| Maternal education (in years) |  |  |  |  |
| 0-4 | 1.00 | 1.00 | 1.00 | 1.00 |
| 5-8 | 0.91 (0.70 - 1.18) | 0.82 (0.62 - 1.09) | 1.20 (0.91 - 1.59) | 1.22 (0.92 – 1.63) |
| 9+ | 0.85 (0.64 - 1.13) | 0.80 (0.59 - 1.09) | 1.08 (0.79 - 1.47) | 1.13 (0.83 – 1.55) |
| Maternal relationship |  |  |  |  |
| Without partner | 1.00 | 1.00 | 1.00 | 1.00 |
| With partner | 0.98 (0.77 – 1.24) | 1.10 (0.86 – 1.40) | 1.00 (0.84 –1.20) | 0.97 (0.81 – 1.16) |
| Income (terciles) |  |  |  |  |
| 1º (poorest) | 1.00 | 1.00 | 1.00 | 1.00 |
| 2º | 0.89 (0.69 - 1.14) | 0.88 (0.69 - 1.12) | 1.00 (0.83 - 1.21) | 0.98 (0.82 – 1.18) |
| 3º (richest) | 0.84 (0.65 - 1.07) | 0.87 (0.67 - 1.14) | 0.90 (0.71 - 1.14) | 0.91 (0.72 – 1.14) |
| Only child |  |  |  |  |
| Yes | 1.00 | 1.00 | 1.00 | 1.00 |
| No | 0.80 (0.65 – 0.99) | 0.91 (0.72 – 1.15) | 0.93 (0.79 – 1.10) | 0.92 (0.78 – 1.09) |
| Overcrowded houses |  |  |  |  |
| < 3 individuals per room | 1.00 | 1.00 | 1.00 | 1.00 |
| ≥ 3 individuals per room | 1.11 (0.89 - 1.37) | 1.04 (0.83 - 1.31) | 1.13 (0.96 - 1.33) | 1.13 (0.96 – 1.33) |
| Time spent with the children during weekdays | |  |  |  |
| < 24h | 1.00 | 1.00 | 1.00 | 1.00 |
| All the time | 0.89 (0.72 - 1.10) | 0.88 (0.72 - 1.09) | 1.13 (0.94 - 1.36) | 1.12 (0.94 – 1.34) |
|  | **Second level** | | | |
| Maternal depression |  |  |  |  |
| No | 1.00 | 1.00 | 1.00 | 1.00 |
| Yes | 1.00 (0.81 - 1.24) | 0.95 (0.76 – 1.20) | 1.02 (0.86 - 1.20) | 0.96 (0.80 – 1.14) |
| Maternal perceived stress |  |  |  |  |
| Low | 1.00 | 1.00 | 1.00 | 1.00 |
| Moderate | 1.13 (0.83 - 1.54) | 1.13 (0.85 – 1.50) | 1.07 (0.88 - 1.29) | 1.06 (0.88 – 1.28) |
| High | 1.05 (0.54 - 2.03) | 0.95 (0.55 – 1.64) | 1.15 (0.82 - 1.63) | 1.16 (0.83 – 1.63) |
|  | **Third level** | | | |
| Maternal intimate partner violence |  |  |  |  |
| Without partner | 1.00 | 1.00 | 1.00 | 1.00 |
| No | 0.98 (0.77 - 1.26) | 1.02 (0.80 - 1.30) | 0.97 (0.79 - 1.18) | 0.96 (0.78 – 1.17) |
| Yes | 0.97 (0.70 - 1.34) | 0.99 (0.72 - 1.35) | 1.07 (0.87 - 1.31) | 1.04 (0.85 – 1.28) |
| Child’s maltreatment |  |  |  |  |
| No | 1.00 | 1.00 | 1.00 | 1.00 |
| Yes | 0.92 (0.67 - 1.25) | 0.86 (0.64 - 1.17) | 0.98 (0.79 - 1.22) | 0.95 (0.76 – 1.19) |
|  | **Fourth level** | | | |
| Positive and involved parenting |  |  |  |  |
| No | **1.00** | 1.00 | 1.00 | 1.00 |
| Yes | **1.25 (1.01 - 1.56)** | 1.18 (0.95 - 1.47) | 1.05 (0.87 - 1.26) | 1.07 (0.88 – 1.28) |
| Harsh parenting |  |  |  |  |
| No | **1.00** | 1.00 | 1.00 | 1.00 |
| Yes | **1.27 (1.04 - 1.56)** | 1.11 (0.89 - 1.38) | 1.12 (0.94 - 1.33) | 1.10 (0.92 – 1.30) |
| Inconsistent discipline |  |  |  |  |
| No | 1.00 | 1.00 | 1.00 | 1.00 |
| Yes | 1.06 (0.82 - 1.38) | 0.95 (0.74 - 1.22) | 0.95 (0.76 - 1.19) | 0.92 (0.73 – 1.15) |
|  | **Fifth level** | | | |
| Participated in PIM |  |  |  |  |
| No | 1.00 | 1.00 | 1.00 | 1.00 |
| Yes | 1.13 (0.90 - 1.41) | 1.04 (0.83 - 1.31) | 0.88 (0.70 - 1.10) | 0.87 (0.70 – 1.08) |
| Participated in any trial |  |  |  |  |
| No | 1.00 | 1.00 | 1.00 | 1.00 |
| Yes | 1.00 (0.78 - 1.28) | 1.05 (0.82 - 1.34) | 1.01 (0.83 - 1.24) | 1.03 (0.84 – 1.26) |
|  | **Sixth level** | | | |
| Child's conduct problems |  |  |  |  |
| Normal | 1.00 | 1.00 | 1.00 | 1.00 |
| High | 1.20 (0.89 - 1.63) | 1.13 (0.84 - 1.51) | 1.13 (0.93 - 1.37) | 1.13 (0.94 – 1.37) |
|  | **Seventh level** | | | |
| Date of the first session |  |  |  |  |
| July | 1.00 | 1.00 | 1.00 | 1.00 |
| August | 0.87 (0.73 – 1.04) | 0.88 (0.75 – 1.04) | 0.86 (0.70 – 1.07) | 1.11 (0.99 – 1.23) |
| September to November | 0.92 (0.80 – 1.07) | 0.98 (0.84 – 1.15) | 1.10 (0.94 – 1.27) | 1.11 (1.00 – 1.22) |
| Perceived distance from DBS/ACT to centre | |  |  |  |
| Far | 1.00 | 1.00 | 1.00 | 1.00 |
| Near | 1.14 (1.00 - 1.30) | 1.05 (0.91 - 1.21) | 1.04 (0.96 - 1.13) | 0.99 (0.93 – 1.06) |
| Distance from DBS/ACT to centre (km) |  |  |  |  |
| ≤1.5 | 1.00 | 1.00 | 1.00 | 1.00 |
| 1.6 – 3.0 | 0.83 (0.64 – 1.08) | 0.86 (0.74 – 1.00) | 0.84 (0.68 – 1.05) | 1.09 (0.99 – 1.20) |
| 3.1 – 5.0 | 0.89 (0.67 – 1.17) | 0.84 (0.70 – 1.00) | 0.98 (0.81 – 1.18) | 1.04 (0.94 – 1.15) |
| >5 | 0.79 (0.55 – 1.15) | 0.79 (0.59 – 1.05) | 0.91 (0.71 – 1.17) | 0.96 (0.82 – 1.13) |
| Satisfaction with the intervention |  |  |  |  |
| Satisfied | 1.00 | **1.00** | **1.00** | **1.00** |
| Very satisfied | 1.14 (0.96 – 1.36) | 1.15 (0.98 – 1.34) | 1.12 (1.01 – 1.25) | 1.11 (1.01 – 1.23) |

95%IC: 95% confidence interval; IRR: incidence rate ratio; alcohol consumption and the category “less than satisfied” were excluded from this analysis due to the small sample sizes
